# A clinical trial of a patient-customized virtual reality intervention for tinnitus

**DOI:** 10.1038/s41598-022-16764-5

**Published:** 2022-07-20

**Authors:** Dong Heun Park, Sang Sun Han, Munsoo Han, Seongbin Park, Hae Nim Kim, Jiyeon Kim, Hojun Aan, Jimoon Kim, Sungkean Kim, Kibum Kim, June Choi

**Affiliations:** 1 Department of Otorhinolaryngology-Head and Neck Surgery, Ansan Hospital, Korea University College of Medicine, 123 Jeokgeum-ro, Danwon-gu, Ansan-si, Gyeonggi-do 15355 Republic of Korea; 2 Department of Human-Computer Interaction, Hanyang University, 55 Hanyangdaehak-ro, Sangnok-gu, Ansan-si, Gyeonggi-do 15588 Republic of Korea; 3 Department of Neurology, Ansan Hospital, Korea University College of Medicine, Ansan, Republic of Korea

**Keywords:** Health care, Medical research

## Abstract

Virtual reality (VR) has recently been used as a clinical treatment because it can efficiently simulate situations that are difficult to control in real-world settings. In our study, we assessed the potential of VR in patients with chronic subjective tinnitus. An evaluation of its clinical benefits was performed based on analyses of patient electroencephalograms (EEGs) and by questionnaire responses after 6–8 weeks of patient involvement in our VR-based alleviation program. Clinical trials were performed at a tertiary academic hospital. Nineteen patients (aged 33–64 years) who visited our hospital with chronic subjective tinnitus over 3 months were enrolled in the study. The intervention consisted of trashing the tinnitus avatar in VR. We expected that the patients would have the subjective feeling of controlling tinnitus through our intervention. The VR environment comprised four different sessions in four different settings: a bedroom, a living room, a restaurant, and a city street. We analyzed changes in the source activities of the prefrontal regions related to tinnitus in these patients using standardized low-resolution brain electromagnetic tomography. The Tinnitus Handicap Inventory (THI), the total score (from 50.11 to 44.21, *P* = 0.046) and the grade (from 3.16 to 2.79, *P* = 0.035) were significantly improved after the VR-based tinnitus treatment program (*P* < 0.05). The Pittsburgh Sleep Quality Index also showed improved outcomes (*P* = 0.025). On the other hand, a Tinnitus Handicap Questionnaire, Quality of Life Assessment (WHO-QOL), Hospital Anxiety and Depression Scale, Profile of Mood States revealed no significant change after the intervention. The baseline EEG data showed that brain activity in the orbitofrontal cortex significantly increased in the alpha and theta frequency bands. Furthermore, patients who showed a THI score improvement after the intervention showed specific increases in brain activity for the theta and high beta bands in the orbitofrontal cortex. Our findings suggest that the virtual reality-based program, as in parts of cognitive behavioral treatment, may help to alleviate tinnitus-related distress in patients with chronic subjective tinnitus.

## Introduction

Tinnitus refers to the subjective feeling of noise from the ear in the absence of external auditory stimuli^1,2^. This condition occurs in 10% of the world's population and severe tinnitus can lead to decreased concentration and to mood disorders which may not only affect the patient’s quality of life but also lead to social and economic losses^3,4^. While the pathophysiologic mechanism for subjective tinnitus remains controversial, a recently described model suggests the hyper-activation and functional reassignment of the auditory and non-auditory cortexes or subcortex networks^5–7^. In other words, subjective tinnitus may be accompanied not only by functional abnormalities in a part of the brain, it may also be accompanied by disrupted brain network connectivity^8,9^.

Various studies have demonstrated that tinnitus originates in the brain rather than in the ear; thus, the monitoring of neuronal activity is needed to describe the mechanism of tinnitus modulation. Analyzing neural behavior at the cortical level, especially alterations of the auditory pathway, helps improve our understanding of neural synchrony and reorganization related to tinnitus development and maintenance.

Recently in South Korea, programs for tinnitus alleviation are being led by professional otolaryngologists, psychologists and audiologists to relieve tinnitus in their patients. This tinnitus alleviating intervention aims to render tinnitus as a natural sound by reducing the association of the symptoms with negative emotions, however this requires considerable time to complete the intervention^10,11^. Since these are also consultation-oriented programs, they may be difficult to understand; and thus, patients often express low interest in them. Hence, it is difficult for patients to obtain completely satisfactory outcomes and results compared to those of other treatments.

The virtual reality (VR) system can provide users with the sense of presence and immersion through 360° visual displays, spatial acoustic sound, and haptic feedback^12^. These realistic user experiences suggest that VR could be a viable clinical treatment method^13^. Recent studies have shown reductions in post-traumatic stress disorder (PTSD) symptoms using VR for the treatment of veterans and active duty service members^14–19^. One study also demonstrated the willingness of military personnel to use VR-based approaches to mental health care^20^. Other studies have reported similar effectiveness between VR and traditional therapies for the treatment of fear of flying, panic disorder with agoraphobia, social phobia, and arachnophobia^21^. Additionally, children with arachnophobia preferred exposure via VR over actual exposure^22^. In our study, we aimed to provide a feeling of control which may lead to the alleviation of tinnitus by allowing patients to manipulate and eliminate objects (VR avatars) which produced tinnitus sounds in a VR environment. In previous work^23,24^, the patients were able to point and navigate the tinnitus avatar. In our experiment, the patients are taking one step further and actually getting rid of the tinnitus avatar by catching and throwing it into the wastebasket. Therefore, the patient felt that the tinnitus could be controlled by erasing the tinnitus avatar.

After the experiment was completed, we evaluated the effectiveness of this VR-based intervention for the treatment of tinnitus patients using questionnaires, electroencephalogram (EEG), and standardized low-resolution brain electromagnetic tomography (sLORETA). The analysis of the EEG data of participants collected before and after the program allowed evaluation of the change factors for the auditory and non-auditory cortexes or subcortex networks. Through this process, we intended to determine the usefulness of VR as a treatment of choice to reduce tinnitus and related symptoms.

## Methods

### Patients and questionnaires

Among patients (aged 19–80 years) who visited our hospital with a chief complaint of tinnitus, we selected those who had chronic non-pulsatile tinnitus for 3 months or longer, were capable of smooth communication in Korean, and who agreed to participate in the study. We excluded those patients with frequent noise exposure as a part of their professional or hobby activities and those who were expected to have difficulty operating a VR program based on a head-mounted display (HMD). Finally, 19 patients (9 male, 10 female) with tinnitus, participated in this study.

The medical history, demographic information, medical examination, and physical examination, including vital signs (blood pressure, heart rate, body temperature, and breathing rate), weight, height, and daily life competency of the participants were evaluated accordingly. Moreover, after confirming the treatment history and underlying diseases related to tinnitus except for other otological, neurological, and psychological problems before the study, audiograms and tinnitus symptoms were also examined for these patients before and after the experiment to exclude unnecessary bias-related cases.

The pre- and post-experimental patient statuses were evaluated through questionnaires on tinnitus itself, including the THI, THQ, and visual numeric scale (VNS) related to the severity of tinnitus-related distress. Although these are not designed as an outcome measure, they are a brief, easily administered, and psychometrically robust measure that evaluates the impact of tinnitus on daily living and widely used in the South Korea health care system^25^. Questionnaires about symptoms associated with tinnitus, such as the PSQI, WHO-QoL, POMS, HADS for depression, anxiety, and sleep disorders accompanying tinnitus, were completed. Further, the simulator sickness questionnaire (SSQ) was administered to evaluate the post-experimental patient symptoms that might occur after usage of the VR system.

### Experimental protocol

**Figure 1:**
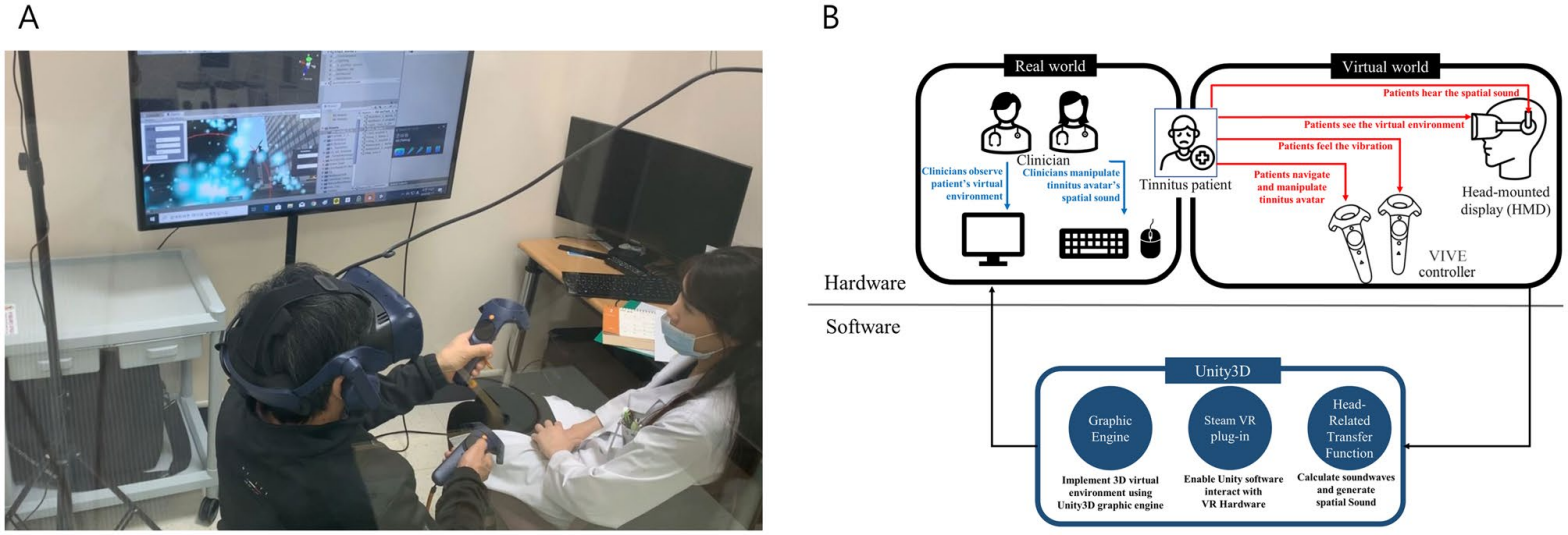
Tinnitus treatment experiment setup. (**A**) The patient and the clinician view the virtual environment through a head-mounted display (HMD) and on a screen, respectively. The patient hears spatial sound from the tinnitus avatar through the HMD headphone. The patient also moves the tinnitus avatar using a VIVE controller. (**B**) Hardware and software setups.

**Figure 2:**
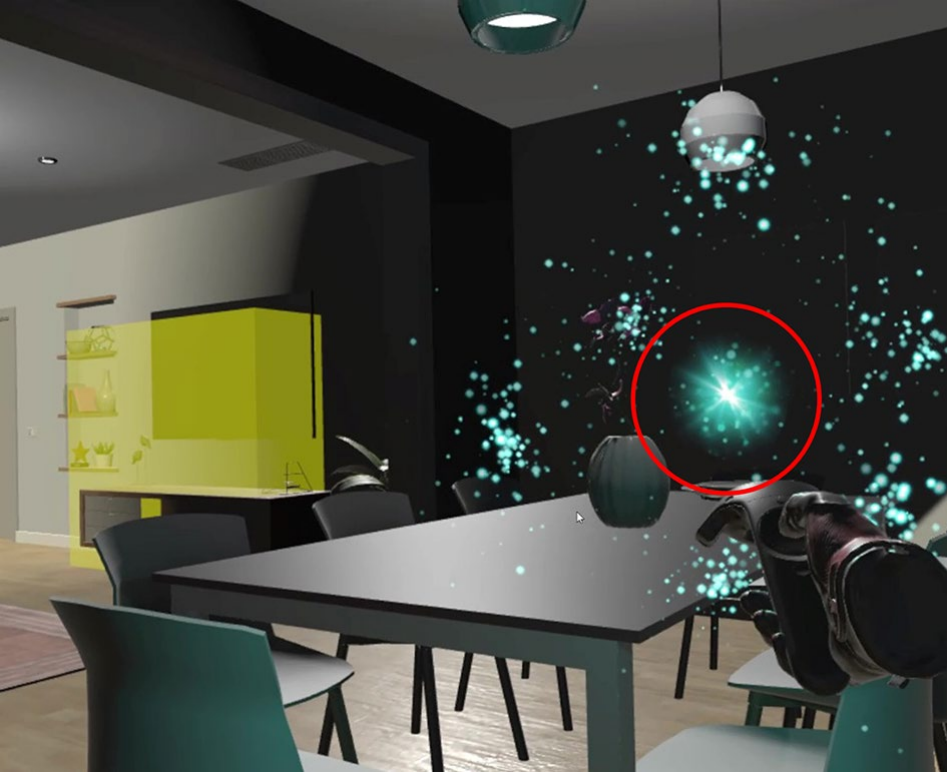
Example of virtual environment for the treatment session. The sparkling object emitting particles (red circle) is a tinnitus avatar. The yellow area is the tinnitus disposal site in which the patients are instructed to discard the tinnitus avatar.

First, a tinnitus avatar was created to mimic the subjective tinnitus of each patient by matching the frequency and loudness. This acoustic modelization of the perceived tinnitus established by the signal is matched to the spectrum and intensity of the tinnitus percept in patients^26,27^. This indicates that a fusion process can occur between subjective tinnitus and the matched stimulus presented in the contralateral ear^23,24^. A tutorial session was conducted before the main treatment session. The tinnitus avatar was designed to produce spatial tinnitus sounds that were implemented with a head-related transfer function (HRTF) of the Google resonance sound software development kit (SDK). The participants learnt how to move in a VR setting and to perform tasks to dispose of the tinnitus avatar (Fig. 1). Using HRTF, the tinnitus avatar produced 3D tinnitus sounds of five types (whistling, hissing, roaring, humming, and a ringing sound). The participants were required to use their hearing to locate the tinnitus avatar. The participants could move in the virtual environment by pushing the upper and lower parts of the trackpad on the left VIVE controller. They were able to see the tinnitus avatar once they had approached it within a certain distance. When the tinnitus avatar was held by pressing the trigger button on the right VIVE controller, the color of the avatar changed and vibration feedback was generated on the right VIVE controller, informing the participants that they had captured the tinnitus avatar. The participants then moved the tinnitus avatar to the tinnitus disposal site in the virtual environment scene (Fig. 2). When the tinnitus avatar was discarded, the tinnitus sound rapidly diminished. For the tinnitus VR intervention sessions, two sets of virtual environment were developed for each city street, restaurant, living room, and bedroom scenes. The tinnitus disposal sites were placed in the noisy scenes in each virtual set (i.e., at the entrance to the bedroom, beside a television in the living room, at the ordering counter in a restaurant, and in a car hood in a city street), creating the cognitive illusion of absorbing tinnitus sounds into much louder environmental noises. Three rounds of tinnitus avatar disposal tasks were performed during each therapy session.

**Figure 3:**
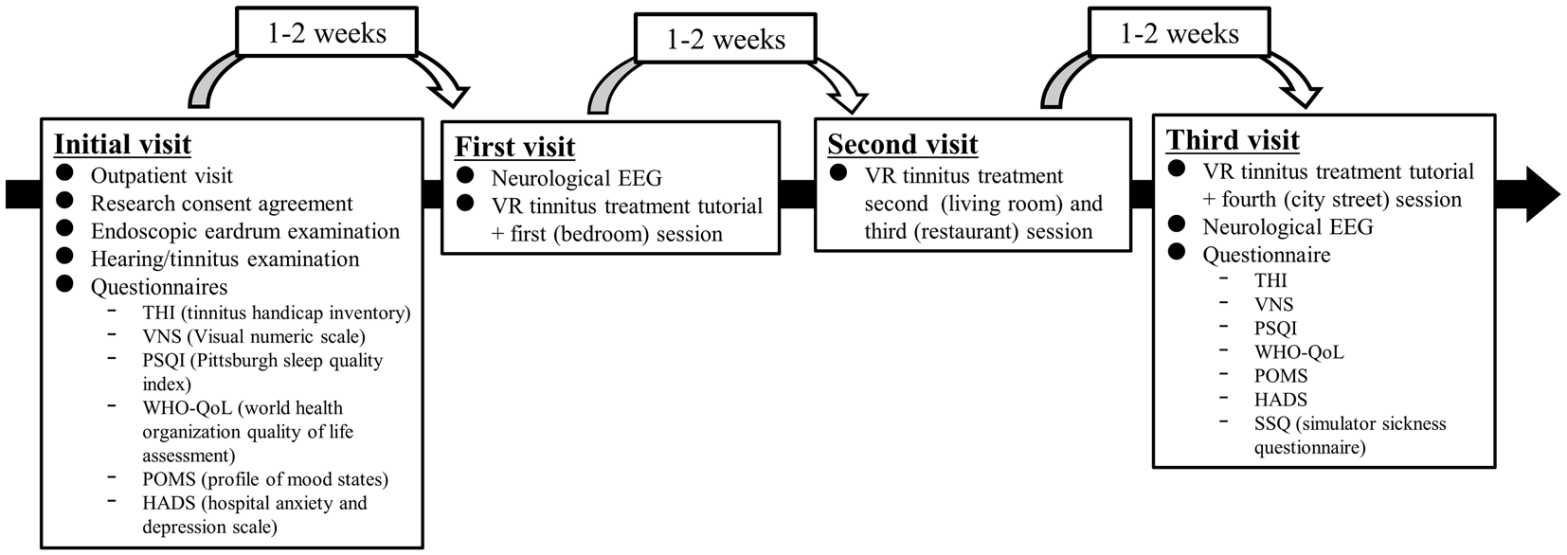
The four stages of the experimental protocol.

**Figure 4:**
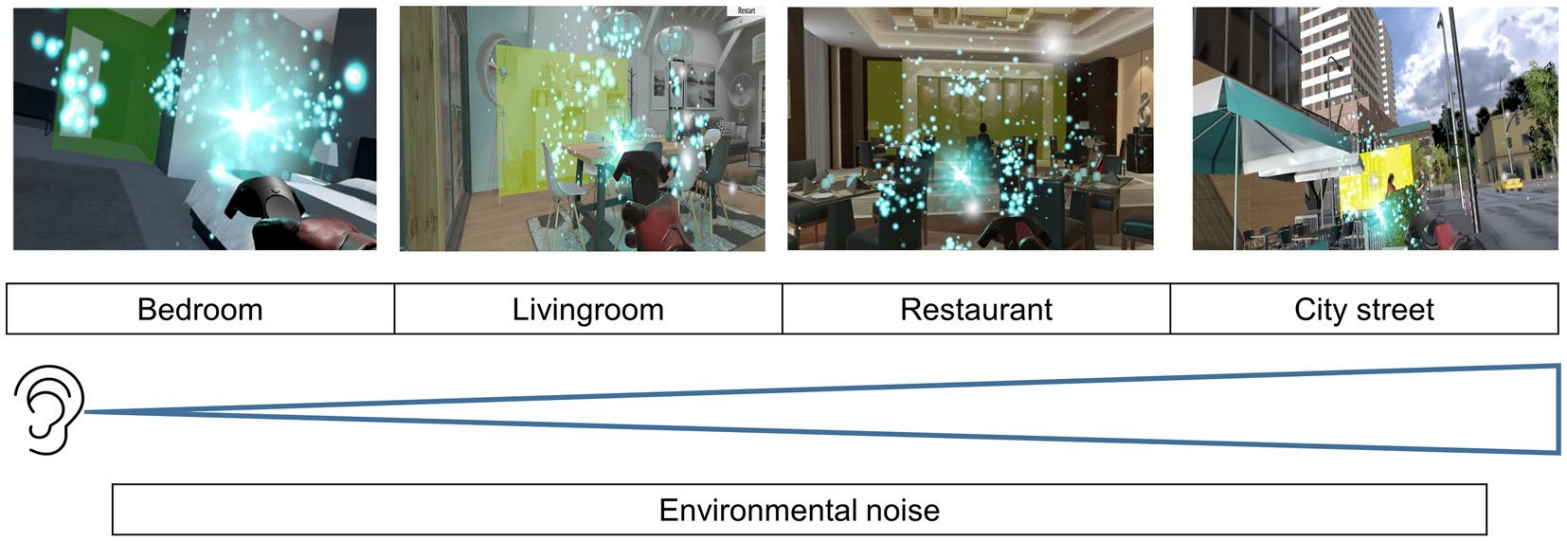
Examples of virtual reality tinnitus treatment scenes. Starting from left to right, the scenes were arranged based on increasing environmental noise. The participants experienced the scenes in order from left to right.

The patients visited the hospital four times and experienced VR tinnitus intervention every 1–2 weeks (Fig. 3). At their initial visit, those who agreed to participate in the experiment underwent an endoscopic eardrum examination and a hearing/tinnitus examination, followed by questionnaires. They experienced the VR tinnitus intervention system from the first to the third visit, for a total of three sessions. During the first visit, they underwent EEG tests (the detailed EEG recording method is described in the supplementary information) to determine the status of their brain waves before experiencing the VR tinnitus intervention system. The first session (city street) was followed by a tutorial session. Participants performed both the second (restaurant scene) and third (living room scene) sessions on their second visit; they performed the fourth session (bedroom scene) on their third visit. The sessions were arranged to decrease the volume of environmental noise with progress through the sequence (Fig. 4). After the fourth session, the participants received an EEG test to and the SSQ questionnaire. Our experimental protocol is summarized in the supplementary video.

### Source localization using sLORETA

We used the sLORETA software which allows the analysis of intracerebral electrical sources from scalp-recorded activity based on EEG data. We preprocessed the EEG data and prepared for 30 epochs per participant, as mentioned in the supplementary information. These epochs were analyzed for six frequency bands (delta, 1–4 Hz; theta, 4–8 Hz; alpha, 8–12 Hz; low beta, 12–18 Hz; high beta, 18–30 Hz; and gamma, 30–55 Hz). In sLORETA, the source images were spatially modeled as a collection of 6239 voxels (size 5 × 5 × 5 mm). These layers were obtained from the amygdala, hippocampus, and cortical gray matter. The sLORETA data were based on the digitized Montreal Neurological Institute (MNI) 152 coordinates reformed to Talairach coordinates^28,29^.

We selected 10 ROIs from prefrontal regions based on the previous literature on tinnitus^5,30–32^. Each ROI comprised one or two Brodmann areas (Table 1). We also compared the current source density for each ROI using SPSS.

**Table 1:** Selected ROIs and BAs.

ROI (Regions of Interest)	BA (Brodmann Areas)
Dorsal anterior cingulate cortex	24L
	24R
Pregenual anterior cingulate	32L
	32R
Subgenual anterior cingulate cortex	25L
	25R
Orbitofrontal cortex	10L&11L
	10R&11R
Dorsolateral prefrontal cortex	9L&46L
	9R&46R

### Outcomes

The primary outcome measure was the questionnaire results of pre- and post-experimental patient status and symptoms associated with tinnitus. The exploratory outcome measure was the current source density for 10 ROIs^5,30–32^. The questionnaire results regarding VR sickness using the SSQ provided another measure.

### Statistical analysis

Descriptive statistics were used to analyze the outcomes. Improvements in the THI score and grade of tinnitus were reported using the change in difference with respect to measurements before treatment. The pre-treatment and post-treatment differences were reported using absolute values. The statistical significance of the differences found in the questionnaire was evaluated using the Wilcoxon signed-rank test. Absolute values before and after treatment were paired for each participant, and statistical significance was considered at p less than 0.05. All data were analyzed using SPSS (version 20.0; SPSS, Chicago, Illinois, USA). Furthermore, we performed a Wilcoxon signed-rank test between EEG data of before-treatment and after-treatment and a Mann–Whitney U test in subgroups separated by THI scores regarding differences in EEG data between before-&-after-treatment on ROI analysis (THI < 0: n = 12, THI ≥ 0: n = 7)^28,29^. In this study, in order to investigate potential EEG indicators which can reflect the improvement of tinnitus-related distress at an exploratory level, we did not perform multiple comparison correction, but considered the significance level as 0.050.

### Ethical approval

The trial was registered at https://cris.nih.go.kr under the registry number KCT0006242 (the full date of first registration, 10/06/2021). The study was approved by the ethics board of the Korea University Ansan Hospital (No. 2020AS0010), and all patients provided written and informed consent. All methods were performed in accordance with relevant guidelines and regulations.

## Results

### Patients demographics and clinical features

Nineteen patients with non-pulsatile tinnitus were selected for this study. The mean participant age was 56.40 (± 8.19) years. The participants had experienced tinnitus symptoms for an average of 7.34 years. 8 patients experienced tinnitus on both sides, 8 on the left side, and 3 on the right side respectively. The average of tinnitus loudness was 5.42 SL (sensation level). All types of tinnitus were studied without exclusion; none of the patients had Meniere’s disease, tympanum-related disease, or other neurological conditions. Patients with psychiatric disorders were excluded from the study (Table 2).

**Table 2:** Patients demographics and clinical features.

Patients (n = 19)	Age (y)	Gender	Tinnitus laterality (Left/Right/Both ears)	Tinnitus duration (y)	Type of Tinnitus	Associated symptoms	Tinnitus Pitch Matching (Hz)	Tinnitus Loudness (SL)
Patient 1	55	F	Both	8	Buzzing	Hearing loss	500	10
Patient 2	62	M	Left	0.3	Ringing	None	4000	5
Patient 3	63	F	Right	5	Ringing	Hearing loss	2000	30
Patient 4	51	M	Left	15	Ocean waves	Hearing loss	4000	0
Patient 5	52	M	Left	2	Ringing	Hearing loss, Dizziness, Ear fullness	6000	5
Patient 6	49	F	Right	1	whooshing	Hearing loss	250	− 5
Patient 7	64	M	Both	10	Crickets	Hearing loss	1000	0
Patient 8	62	F	Left	13	static	Hearing loss, Dizziness	8000	10
Patient 9	58	F	Both	3	Crickets	Hearing loss	6000	0
Patient 10	58	F	Both	10	whooshing	None	8000	5
Patient 11	43	F	Both	1	Buzzing	Hearing loss, Dizziness	4000	0
Patient 12	63	M	Both	5	Crickets	Hearing loss	8000	− 5
Patient 13	64	F	Left	13	Ocean waves	None	2000	− 10
Patient 14	58	M	Left	0.25	Ocean waves	Hearing loss	8000	− 5
Patient 15	53	F	Both	10	Ringing	Dizziness	8000	5
Patient 16	57	F	Both	7	Electrical	Hearing loss, Dizziness	8000	5
Patient 17	62	M	Left	30	Buzzing	Hearing loss, Dizziness, Ear fullness	4000	5
Patient 18	64	M	Left	1	Ringing	None	8000	0
Patient 19	33	M	Right	5	Dial tones	Hearing loss	4000	20
Mean ± SD	56.4 ± 8.19			7.34 ± 7.23				5.42 ± 9.22

### Analysis of the questionnaires administered to the study participants

**Figure 5:**
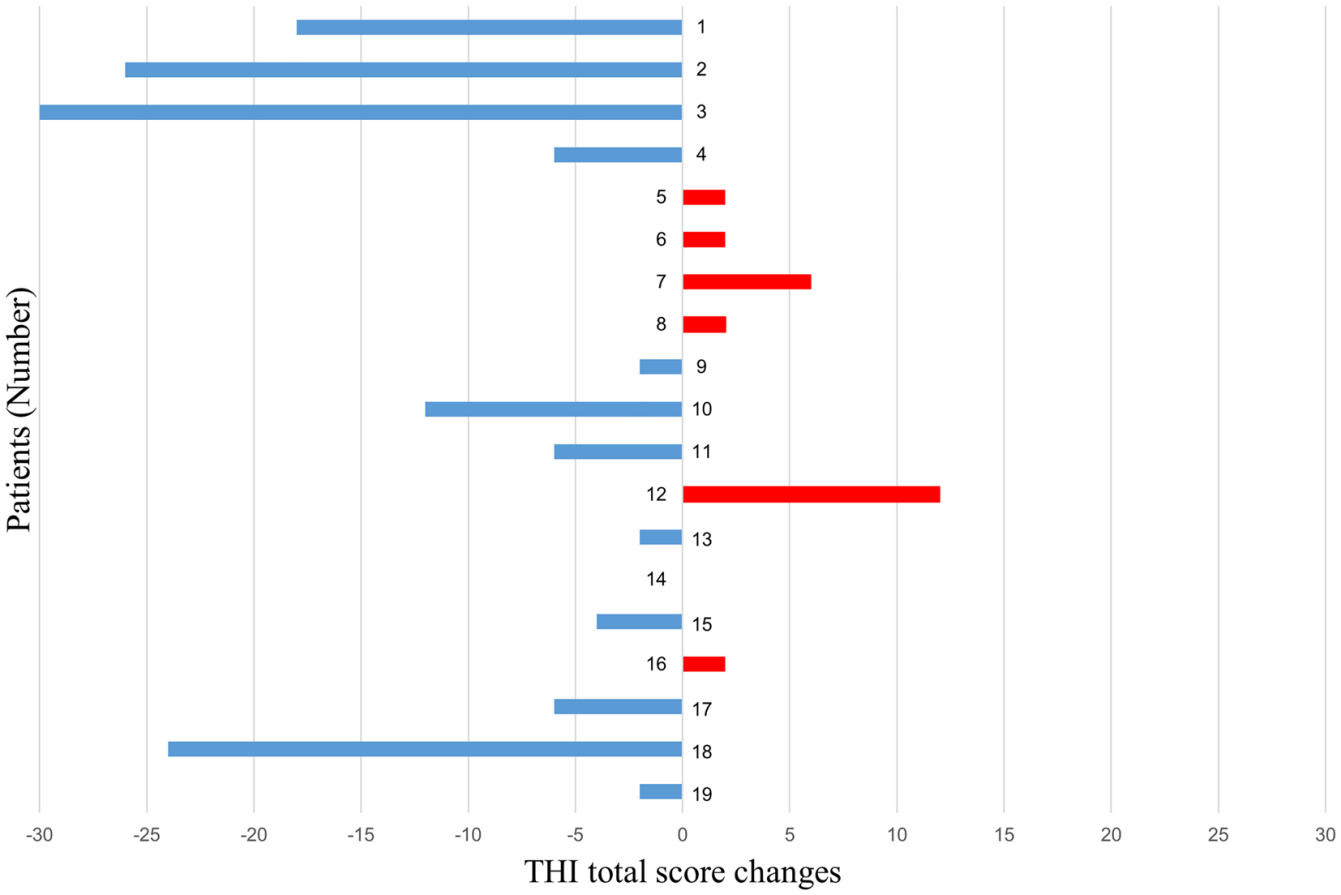
Changes in THI total score. 12 of 19 Patients showed improvement in THI total score (THI, Tinnitus Handicap Inventory).

The participants completed the same tinnitus-related questionnaire before and after their participation in the VR program. Tinnitus improved following treatment based on the THI and PSQI, which are closely related to tinnitus. Statistically significant differences were observed for the THI functional score (*P* = 0.005), total score (*P* = 0.046), and grade (*P* = 0.035) (Table 3). Figure 5 shows the tinnitus-relieving effects according to individual total THI score changes before and after the program. Unlike statistically significant change, clinically meaningful changes been defined as comprising at least 7 points for the THI^33^. In our sample, 6 out of 19 patients met this threshold (37%). But there was no correlation between the THI score and PSQI in the supplementary information. Other questionnaires which are tools for the evaluation of symptoms related to tinnitus, did not show any statistically significant differences.

**Table 3:** Results of the questionnaires administered to the study participants.

	Before VR	After VR	Difference	*P* value
**THI**				
Functional scale	22.00	17.00	− 5.00	0.005
Emotional scale	19.00	16.70	− 2.30	0.108
Catastrophic scale	10.00	10.00	0	0.968
Total	50.11	44.21	− 5.90	0.046
Grade	3.16	2.79	− 0.37	0.035
**PSQI**	8.47	7.37	− 1.10	0.025
**THQ**				
Somatic score	49.40	44.45	− 4.95	0.198
Emotional score	41.00	38.00	− 3.00	0.257
Social score	60.00	60.00	0	0.727
Total	48.50	45.30	− 3.20	0.165
**QOL**	72.63	73.84	+ 1.21	0.434
**HAD**				
Depression	7.58	7.74	+ 0.16	0.916
Anxiety	7.89	7.79	− 0.10	0.354
**POMS**	81.32	77.79	+ 3.53	0.778

THI: Tinnitus Handicap Inventory; PSQI: Pittsburgh Sleep Quality Index; THQ: Tinnitus Handicap Questionnaire; WHO-QoL: World Health Organization Quality of Life assessment; HADS: Hospital Anxiety and Depression Scale; POMS: Profile of Mood States.

For SSQ^34^, the total score was calculated using the following formula: Total score = nausea score + oculomotor score + disorientation × 3.74). The nausea, oculomotor, disorientation, and total scores for our system were 48.20, 58.25, and 74.73, respectively (Table 4). After applying the weights, the total score was 32.81 points.

**Table 4:** Analysis of nausea, oculomotor, disorientation, and total score derived from the SSQ.

SSQ (Simulator Sickness Questionnaire)			
Nausea (sd)	Oculomotor(sd)	Disorientation(sd)	Total Score(sd)
48.20 (33.97)	58.25 (36.81)	74.73 (60.63)	32.81 (24.03)

sd: standard deviation.

### Analysis of EEG data

**Figure 6:**
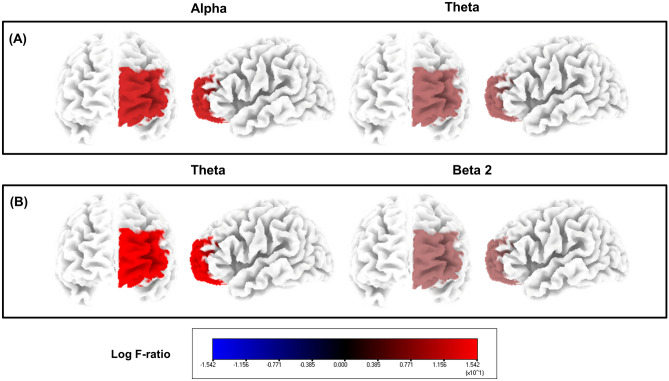
Source-localized cortical power differences in the prefrontal cortex. Increased power levels in the alpha and theta frequency bands (**A**) and theta and beta 2 frequency bands (**B**) after virtual reality (VR) tinnitus treatment.

Compared to the EEG data of pre-VR-based tinnitus intervention, the EEG data of post-VR were analyzed based on the results of the source localization analysis, and the ROIs of the prefrontal cortex were compared accordingly. Significant differences were observed in the ROIs of the orbitofrontal cortex (OFC). The results showed that alpha (*P* = 0.030) and theta (*P* = 0.040) bands were significantly increased in the left orbitofrontal cortex (OFC, 10 L and 11 L) for all patients after VR treatment programs (Fig. 6). Specifically, in the patient group with improved THI scores, significant increases in theta (*P* = 0.003) and high beta (*P* = 0.005) were confirmed in the left OFC (10 L and 11 L).

Additionally, a comparative evaluation was performed between the patient group with improved THI score and the group with no improved THI score regarding the change in each band. The results showed statistically significant differences in theta (*P* = 0.007) and high beta (*P* = 0.001) bands in the left OFC (10 L and 11 L) in the patient subgroups separated based on THI score (THI < Grade 2, THI > Grade 3).

Also, a nonparametric correlation analysis was performed in all patient groups to determine whether the changes of the THI score correlated with the changes in the source activity of specific bands and it was confirmed that there was a significant negative correlation between the change in the THI score and the increase in the high beta band in the left OFC (r = − 0.627, *P* = 0.004). The theta band was analyzed using the same process, and it was confirmed that there was a significant correlation between the THI score and the theta band value in the left OFC (r = − 0.490, *P* = 0.033). In detailed analysis of the subscales of THI, we found that there is a significant correlation between the THI emotional score and the theta value of the left OFC (r = − 0.494, *P* = 0.032). We recorded detailed information of a correlation between the THI scores (including all subscales) and all significant EEG values in the supplementary information.

Additionally, the patient group with improved THI score, the alpha (*P* = 0.028), low beta (*P* = 0.012), and gamma (*P* = 0.015) bands in the left OFC region were significant after our treatment program. Similar changes were found in the right OFC region in the alpha (*P* = 0.041) and the theta (*P* = 0.023) bands. We also observed changes in the group of patients who conducted the experiment in the left sgACC region in a subgroup with improved THI. As a result of our analysis in the patient group with improved THI, theta (*P* = 0.023) and gamma (*P* = 0.028) bands tended to increase in this region accordingly.

## Discussion

Using a VR system with high visual, auditory, and tactile reality, this study evaluated the value of VR-based psychological intervention which forms part of behavioral cognitive therapy for tinnitus patients. We started with the assumption that this method can be used in the recovery of patients with severe tinnitus. Our analysis of the questionnaires showed that VR-based intervention relieved tinnitus and related symptoms. The THI and PSQI scores showed significant improvements, especially in the total score, grade, and functional states. The THI, which is useful for measuring severity of tinnitus-related distress and predicting psychological distress, varies across individuals but scores improved in 12 patients of 19 total patients^25^. The PSQI, which indicates the degree of sleep quality, was also lower after the intervention, showing that the program helped to relieve related symptoms such as insomnia induced by severe tinnitus. On the other hand, the reason that the other questionnaires did not show a statistically significant difference is that they evaluate indirect factors related to tinnitus and the change may have been small due to the short treatment period.

Our VR system showed relatively low levels of motion sickness based on the analysis of the SSQ. In the previous research, the SSQ score of the VR therapy system for the chronic pain patient 55.72^26^ and the SSQ score of the VR wheelchair training simulator system was above 200^27^. On the other hand, the SSQ score of our VR system was 32.81. The lower SSQ score indicated less motion sickness. The lower SSQ score of our VR system indicated that the system itself did not create the issue of motion sickness. We also carefully analyzed the EEG data before and after the experiment. The corresponding analysis confirmed that the source activities of the alpha and theta bands were increased in the left orbitofrontal cortex in all patient groups after VR treatment programs.

Generally, limbic systems are highly relevant to acoustic features, such as tinnitus and phantom sounds. This is due to interactions between the limbic and auditory systems, which may be related to the noise-canceling system^5^. This system is closely connected to tinnitus-induced stress, which is caused by corresponding changes in the prefrontal cortical system of the brain^31,35^. Recently, in an integrative model of tinnitus suggesting tinnitus as a unified perception of interacting divided subnetworks, the orbitofrontal cortex (OFC) was proposed as a cortical area responsible for the emotional component of tinnitus. Particularly, areas such as these play a role in orienting attentional and emotional modulation to inhibit unwanted sensory signals while passing through the thalamus and nucleus accumbens. Moreover, mild structural and functional abnormalities of the PFC are often observed in tinnitus patients. Recent studies have also shown that OFC positively correlated with percentage improvements in numeric rating scale (NRS) distress and changes in the source-localized cortical power in the OFC for the alpha frequency band, which is linked to excitatory rather than inhibitory activities regarding the feeling of pleasantness correlated with this cortical area^36^. Further, similar to these studies, our EEG results also confirmed the connection between the alpha band in the OFC and tinnitus. After our customized VR intervention, the patient’s EEG results showed an increased source activity of the alpha band, indicating an increased excitatory function and, thus, proposed as a restoration of the emotional regulation system, which is related to the development of tinnitus.

We also observed increased theta activity in the OFC. Previous studies demonstrated that source localization analysis of theta changes in tinnitus improvement groups through musical therapy found that primary sources of the changes started in auditory processing regions such as the superior temporal gyrus, and higher emotional and cognitive processing regions such as the OFC^37^. Also, abnormalities and theta activity changes in the limbic system regions are frequently observed in tinnitus patients, interpreted as retrieval of auditory information from memory as a compensation for insufficient sensory input to alleviate uncertainty, which can cause an accumulation of emotional memory associated with tinnitus^38^. Furthermore, previous findings have shown that the OFC, which also plays a role in control and working memory function to initiate a task, has connectivity to the limbic system that may be expressed as increased theta activities^39,40^. Therefore, our results in the tinnitus patients involved in our study could be evidence of improved cognitive control, especially inhibitory executive control in multisensory tasks and in the memory process. This may be caused by phase-locked theta activities that correlated with this region in top-down task performance and improvement of cognitive control for tinnitus patients due to the alleviation of symptoms by our VR program^41^.

We performed further analyses on the patient group with improved THI scores, and significant increases in theta and high beta were confirmed in the left OFC. Additionally, a comparative evaluation was performed, and the results showed statistically significant differences in the theta and high beta bands in the left OFC.

The beta band also plays a role similar to the alpha band in the OFC region related to the emotional system. As suggested above, the beta band is closely related to the unpleasant feelings of tinnitus. As such, the beta band is an expression of the changes in distress that are perceived by a network consisting of the limbic system, which activates the anterior cingulate, amygdala, and insula to the prefrontal cortex system^42^. Beta bands increases, as in our results, might activate the functional connectivity between the precuneus and the OFC and dorsolateral prefrontal cortex (DLPFC), which is related to emotional processing, and which in turn is closely related to the tinnitus alleviation system^30,43^. This could orient attentional and emotional modulation to inhibit and alleviate the irritation from tinnitus, as in other reports^42^.

Additionally, in the patient group with improved THI score, the alpha, low beta, and gamma bands in the left OFC region were not significant, however they showed a tendency to increase after our treatment program. These results consequently suggest that additional factors were discovered through a more detailed analysis, as described above, and also suggest consequent correspondence to the increased alpha band in the left OFC region, which was seen in all patient groups. Similar changes were found in the right OFC region in the alpha and theta bands. We also observed changes in the group of patients who participated in the experiment in the left sgACC region in a subgroup with reduced THI. The sgACC area closely regulates positive emotion, the arousal processing network and error detection function and it is also involved in the adverse effects caused by tinnitus and similar disorders such as chronic pain and post-traumatic stress disorder^44,45^. Another study reported that a higher activity of the ACC predicts a higher level of tinnitus distress felt by the patient^46–49^. Owing to our analysis in the patient group with improved THI, theta and gamma bands tended to increase in this region, which may correlate with restoration of the processing system.

Furthermore, it was confirmed through nonparametric correlation analysis in all patient groups that the changes in the THI score after experiencing the VR treatment program correlated with the changes in the source activity of specific bands, and it was confirmed that there was a significant negative correlation (r = − 0.627) between the change in the THI score and an increase in the high beta band in the OFC. That is, when the high beta band value increased, the THI score improved in the patient. Additionally, the theta band was analyzed through the same process, and it was confirmed that there was a significant correlation between the THI score and theta band value (r = − 0.490) in the OFC. These results demonstrate that the VR intervention program conducted through our experiment is effective in clinically improving tinnitus-related distress through correlation of the THI score and this is verified by the changes in the brain signal through the EEG. Our study provides an understanding of intervention-related changes which seem to involve cortical regions pertaining to central representations of tinnitus-related distress.

VR has recently begun to permeate our lives with the emergence of various devices and research related to VR. This study evaluated the functional differences in the brain cortex related to tinnitus after exposure to a VR program and verified that the program induced changes at the brain level, especially in the OFC and the sgACC areas. These changes may lead to the remodulation of the disturbed network in related regions and they may influence the modulation of the disrupted pathways^50^. The VR program in the present study allowed the participants to visualize auditory stimulation, which caused a change in the OFC and sgACC mechanism that processed and modulated tinnitus, leading to decreased tinnitus. In other words, the roles of these areas, which are related to cognition, mood, and affection, are closely related to instances of tinnitus and its effects and this could be a clue to the basic pathophysiology of tinnitus. Moreover, the band differences shown in the EEG indicate the complementary network activation of these functions. Thus, cognitive behavioral therapy may induce changes in tinnitus generation and the ongoing experience of tinnitus.

However, our study has several limitations. First, the number of patients was small and the treatment period was short; thus, the functional difference induced by cognitive behavioral therapy was not significant. While we believe that an increased 'sense of control might' mediate the method's effect on tinnitus-related distress, future studies will have to measure and examine putative psychological processes that underlie the observed effect. Additionally, several participants showed reduced performance since VR systems have not been popularized in the general public. There was also a limitation in that the VR system provided only two scenes for each training environment. VR-based treatments can be improved by providing more scenes and increasing patient exposure to virtual reality. Third, there is no control group because these interventions could not be applied to patients without tinnitus. Although a control-group design could not involve individuals without tinnitus, future studies might wish to examine the intervention's effect for patients with chronic tinnitus vs. other somatization phenomena or functional audiological presentations such as medically unexplained migraines, vertigo, hyperacusis, etc. In addition, statistically significant change does not necessarily imply clinically meaningful change. Future studies might wish to maximize factors that predict clinically significant change in our patient population. Also, as a preliminary research, no multiple comparison correction factor is employed in this study but a more conservative approach might require further study. Finally, each patient had a different hearing ability, which could cause a bias in the brain cortex level owing to the tinnitus mechanism gap between individuals with normal hearing and those with hearing loss. Despite these limitations, we expect this study to become a stepping-stone towards improved methods for VR-based treatments of tinnitus patients.

## Conclusion

Chronic subjective tinnitus affects the patient’s life in various bad ways and, so far, there is no truly successful treatment. Among available treatments, cognitive behavior therapy is recently in the spotlight. But there are limitations that need much attention and compliance by patients. Thus, our research team seeks to assess the potential of VR intervention systems that can provide users with realistic experiences which can also be part of treatment in cognitive behavior therapy. We detected relief of tinnitus symptoms and changes in EEG patterns that relate to the tinnitus generating system. However, due to time and material limitations, intervention did not produce the big changes that we expected. If we overcome the limitations mentioned in the discussion we may find a new way to treat tinnitus patients.

## Supplementary Information

Supplementary Video 1.

Supplementary Information.

## Data Availability

The datasets generated and/or analysed during the current study are not publicly available, but are available from the corresponding author on reasonable request.
