# Supplementary material for: A clinical trial of a patient-customized virtual reality intervention for tinnitus
Source: Sci Rep. 2022 Jul 20;12:12441. doi: 10.1038/s41598-022-16764-5 (PMC9300604; doi:10.1038/s41598-022-16764-5)
Supplement: Supplementary file 2 — Supplementary Information. [file 41598_2022_16764_MOESM2_ESM.docx]

**Supplementary Information**

**EEG recordings**

EEG measurements were performed using EEG examination equipment (Digital 40 ch Comet GRASS) owned by the Department of Neurology at our hospital. These two measurements were taken at the beginning and end of the study to evaluate changes facilitated by the participants’ experiences with the VR tinnitus alleviation system. The resting-state EEG, in a stable state with eyes closed for 3 min, was recorded using 19 channels at a sampling rate of 200 Hz with a frequency band ranging from 0.1 Hz to 70 Hz. The ground channel was located under the left chin, and the reference channel was placed between the FP1 and FP2 channels. Twenty-one electrodes were placed according to the International 10–20 EEG system, including the standard 16 temporal and parasagittal scalp sites and Fz, Cz, Pz, A1, and A2. Artifact identification was used, including two sites near the eyes, plus respiration and electrocardiography recordings. The impedance did not exceed 5 kΩ. The EEG data were preprocessed using EEGLAB (version 2021) in MATLAB. The EEG data were re-referenced based on the average reference. Baseline correction was performed for all channels. Band pass filtering was filtered from 1 to 55 Hz. After splitting the pre-processed EEG data by 2s epochs, artifacts were removed with ICA analysis and visual inspection by an EEG researcher. Moreover, all epochs, including amplitudes exceeding ± 75uV at any of the 19 electrodes, were rejected. Finally, 30 artifact-free epochs were set for each participant.

**Video. Summarized experimental protocol.**

Subtitle in video

We developed a serious virtual reality game to relieve the symptoms of tinnitus patients. In our system, the patient could play with tinnitus in a virtual environment using a VR controller. This lighting object is a tinnitus avatar that produces a tinnitus sound, and the yellow box is the goalbox that the patient can use to eliminate the tinnitus avatar. If the patient put the tinnitus avatar into the goalbox, the tinnitus avatar is removed along with tinnitus sound. Therefore, the patient feels that the tinnitus can be controlled by erasing the tinnitus avatar. Now, let us watch the experimental video of our system.

Researcher: Let’s catch the tinnitus avatar.

Researcher: Then throw it away into the box.

Researcher: Let’s try one more time.

Thank you for watching

**Correlation matrix between the obtained questionnaire scores**

|  | | PSQI Variance | THI Variance | THI Variance  (Catastrophic subscale) | THI Variance  (Functional subscale) | THI Variance  (Emotional subscale) |
| --- | --- | --- | --- | --- | --- | --- |
| PSQI Variance | correlation  coefficient | 1.000 | -0.022 | 0.035 | -0.169 | -0.042 |
|  | Probability |  | 0.929 | 0.886 | 0.490 | 0.864 |
|  | N | 19 | 19 | 19 | 19 | 19 |

**Correlation matrix for both the EEG and questionnaire values**

All patient /Left OFC, 10&11L

| Theta/THI total score | THI-Baseline | THI-Changed Score |
| --- | --- | --- |
| EEG-Baseline | r = -0.358, p=0.133 | r = -0.206, p=0.398 |
| EEG-Changed Score | r = -0.047, p=0.850 | r = -0.069, p=0.780 |

| Theta/THI F score | THI-Baseline | THI-Changed Score |
| --- | --- | --- |
| EEG-Baseline | r = -0.431, p=0.065 | r = -0.183, p=0.452 |
| EEG-Changed Score | r = -0.186, p=0.445 | r = -0.086, p=0.725 |

| Theta/THI E score | THI-Baseline | THI-Changed Score |
| --- | --- | --- |
| EEG-Baseline | r = -0.494, P=0.032* | r = -0.374, p=0.115 |
| EEG-Changed Score | r = -0.219, p=0.367 | r = -0.125, p=0.609 |

| Theta/THI C score | THI-Baseline | THI-Changed Score |
| --- | --- | --- |
| EEG-Baseline | r = -0.191, p=0.434 | r = -0.167, p=0.493 |
| EEG-Changed Score | r = 0.152, p=0.535 | r = 0.071, p=0.774 |

| High Beta/THI total score | THI-Baseline | THI-Changed Score |
| --- | --- | --- |
| EEG-Baseline | r = 0.008, p=0.974 | r = 0.177, p=0.469 |
| EEG-Changed Score | r = 0.062, p=0.800 | r = 0.069, p=0.777 |

| High Beta/THI F score | THI-Baseline | THI-Changed Score |
| --- | --- | --- |
| EEG-Baseline | r = -0.037, p=0.881 | r = 0.110, p=0.653 |
| EEG-Changed Score | r = 0.010, p=0.969 | r = -0.011, p=0.966 |

| High Beta/THI E score | THI-Baseline | THI-Changed Score |
| --- | --- | --- |
| EEG-Baseline | r = -0.077, p=0.755 | r = 0.080, p=0.744 |
| EEG-Changed Score | r = -0.064, p=0.794 | r = 0.066, p=0.788 |

| High Beta/THI C score | THI-Baseline | THI-Changed Score |
| --- | --- | --- |
| EEG-Baseline | r = 0.040, p=0.872 | r = 0.232, p=0.339 |
| EEG-Changed Score | r = 0.162, p=0.506 | r = 0.247, p=0.308 |

Patient group with improved THI score/Left OFC, 10&11L

| Alpha/THI total score | THI-Baseline | THI-Changed Score |
| --- | --- | --- |
| EEG-Baseline | r = -0.305, p=0.335 | r = -0.025, p=0.940 |
| EEG-Changed Score | r = -0.344, p=0.274 | r = -0.077, p=0.812 |

| Alpha/THI F score | THI-Baseline | THI-Changed Score |
| --- | --- | --- |
| EEG-Baseline | r = -0.434, p=0.159 | r = -0.004, p=0.991 |
| EEG-Changed Score | r = -0.427, p=0.167 | r = -0.120, p=0.710 |

| Alpha/THI E score | THI-Baseline | THI-Changed Score |
| --- | --- | --- |
| EEG-Baseline | r = -0.470, p=0.123 | r = -0.148, p=0.568 |
| EEG-Changed Score | r = -0.505, p=0.094 | r = -0.205, p=0.523 |

| Alpha/THI C score | THI-Baseline | THI-Changed Score |
| --- | --- | --- |
| EEG-Baseline | r = -0.092, p=0.776 | r = 0.000, p=1.000 |
| EEG-Changed Score | r = -0.099, p=0.760 | r = 0.086, p=0.790 |

| Low Beta/THI total score | THI-Baseline | THI-Changed Score |
| --- | --- | --- |
| EEG-Baseline | r = -0.186, p=0.563 | r = 0.105, p=0.745 |
| EEG-Changed Score | r = -0.067, p=0.837 | r = 0.182, p=0.571 |

| Low Beta/THI F score | THI-Baseline | THI-Changed Score |
| --- | --- | --- |
| EEG-Baseline | r = -0.350, p=0.265 | r = 0.095, p=0.768 |
| EEG-Changed Score | r = -0.224, p=0.484 | r = 0.039, p=0.905 |

| Low Beta/THI E score | THI-Baseline | THI-Changed Score |
| --- | --- | --- |
| EEG-Baseline | r = -0.319, p=0.312 | r = -0.039, p=0.905 |
| EEG-Changed Score | r = -0.235, p=0.462 | r = 0.088, p=0.785 |

| Low Beta/THI C score | THI-Baseline | THI-Changed Score |
| --- | --- | --- |
| EEG-Baseline | r = -0.014, p=0.965 | r = 0.054, p=0.868 |
| EEG-Changed Score | r = 0.198, p=0.538 | r = 0.338, p=0.283 |

| Gamma/THI total score | THI-Baseline | THI-Changed Score |
| --- | --- | --- |
| EEG-Baseline | r = 0.239, p=0.455 | r = 0.165, p=0.609 |
| EEG-Changed Score | r = 0.049, p=0.879 | r = 0.049, p=0.880 |

| Gamma/THI F score | THI-Baseline | THI-Changed Score |
| --- | --- | --- |
| EEG-Baseline | r = 0.238, p=0.457 | r = -0.018, p=0.957 |
| EEG-Changed Score | r = 0.035, p=0.914 | r = -0.148, p=0.646 |

| Gamma/THI E score | THI-Baseline | THI-Changed Score |
| --- | --- | --- |
| EEG-Baseline | r = 0.088, p=0.786 | r = 0.247, p=0.438 |
| EEG-Changed Score | r = -0.228, p=0.476 | r = 0.011, p=0.974 |

| Gamma/THI C score | THI-Baseline | THI-Changed Score |
| --- | --- | --- |
| EEG-Baseline | r = 0.057, p=0.861 | r = 0.262, p=0.410 |
| EEG-Changed Score | r = 0.099, p=0.760 | r = 0.208, p=0.516 |

Patient group with improved THI score/Right OFC, 10&11R

| Theta/THI total score | THI-Baseline | THI-Changed Score |
| --- | --- | --- |
| EEG-Baseline | r = -0.154, p=0.632 | r = -0.035, p=0.914 |
| EEG-Changed Score | r = -0.312, p=0.323 | r = -0.091, p=0.778 |

| Theta/THI F score | THI-Baseline | THI-Changed Score |
| --- | --- | --- |
| EEG-Baseline | r = -0.420, p=0.175 | r = -0.102, p=0.752 |
| EEG-Changed Score | r = -0.483, p=0.112 | r = -0.152, p=0.638 |

| Theta/THI E score | THI-Baseline | THI-Changed Score |
| --- | --- | --- |
| EEG-Baseline | r = -0.232, p=0.469 | r = -0.088, p=0.785 |
| EEG-Changed Score | r = -0.382, p=0.220 | r = -0.198, p=0.538 |

| Theta/THI C score | THI-Baseline | THI-Changed Score |
| --- | --- | --- |
| EEG-Baseline | r = 0.102, p=0.751 | r = 0.086, p=0.790 |
| EEG-Changed Score | r = -0.025, p=0.939 | r = 0.101, p=0.756 |

| Alpha/THI total score | THI-Baseline | THI-Changed Score |
| --- | --- | --- |
| EEG-Baseline | r = -0.284, p=0.371 | r = 0.021, p=0.948 |
| EEG-Changed Score | r = -0.204, p=0.526 | r = 0.056, p=0.863 |

| Alpha/THI F score | THI-Baseline | THI-Changed Score |
| --- | --- | --- |
| EEG-Baseline | r = -0.420, p=0.175 | r = 0.053, p=0.870 |
| EEG-Changed Score | r = -0.371, p=0.236 | r = -0.025, p=0.939 |

| Alpha/THI E score | THI-Baseline | THI-Changed Score |
| --- | --- | --- |
| EEG-Baseline | r = -0.421, p=0.173 | r = -0.134, p=0.677 |
| EEG-Changed Score | r = -0.323, p=0.306 | r = -0.032, p=0.922 |

| Alpha/THI C score | THI-Baseline | THI-Changed Score |
| --- | --- | --- |
| EEG-Baseline | r = -0.081, p=0.802 | r = 0.043, p=0.894 |
| EEG-Changed Score | r = 0.110, p=0.735 | r = 0.287, p=0.365 |

Patient group with improved THI score/Left sgACC, 25L

| Theta/THI total score | THI-Baseline | THI-Changed Score |
| --- | --- | --- |
| EEG-Baseline | r = -0.319, p=0.312 | r = -0.084, p=0.795 |
| EEG-Changed Score | r = -0.218, p=0.497 | r = 0.042, p=0.897 |

| Theta/THI F score | THI-Baseline | THI-Changed Score |
| --- | --- | --- |
| EEG-Baseline | r = -0.448, p=0.145 | r = -0.166, p=0.607 |
| EEG-Changed Score | r = -0.392, p=0.208 | r = -0.018, p=0.957 |

| Theta/THI E score | THI-Baseline | THI-Changed Score |
| --- | --- | --- |
| EEG-Baseline | r = -0.505, p=0.094 | r = -0.230, p=0.473 |
| EEG-Changed Score | r = -0.323, p=0.306 | r = -0.071, p=0.827 |

| Theta/THI C score | THI-Baseline | THI-Changed Score |
| --- | --- | --- |
| EEG-Baseline | r = -0.028, p=0.931 | r = 0.090, p=0.781 |
| EEG-Changed Score | r = -0.021, p=0.948 | r = 0.180, p=0.576 |

| Gamma/THI total score | THI-Baseline | THI-Changed Score |
| --- | --- | --- |
| EEG-Baseline | r = -0.158, p=0.624 | r = -0.158, p=0.625 |
| EEG-Changed Score | r = 0.042, p=0.897 | r = -0.025, p=0.940 |

| Gamma/THI F score | THI-Baseline | THI-Changed Score |
| --- | --- | --- |
| EEG-Baseline | r = -0.147, p=0.649 | r = -0.074, p=0.819 |
| EEG-Changed Score | r = 0.140, p=0.665 | r = 0.011, p=0.974 |

| Gamma/THI E score | THI-Baseline | THI-Changed Score |
| --- | --- | --- |
| EEG-Baseline | r = -0.063, p=0.845 | r = -0.092, p=0.776 |
| EEG-Changed Score | r = 0.042, p=0.897 | r = 0.049, p=0.879 |

| Gamma/THI C score | THI-Baseline | THI-Changed Score |
| --- | --- | --- |
| EEG-Baseline | r = -0.474, p=0.120 | r = -0.122, p=0.705 |
| EEG-Changed Score | r = -0.300, p=0.343 | r = 0.043, p=0.894 |
